# Supplementary material for: Characterization of Inorganic Scintillator Detectors for Dosimetry in Image-Guided Small Animal Radiotherapy Platforms
Source: Cancers (Basel). 2023 Feb 3;15(3):987. doi: 10.3390/cancers15030987 (PMC9913621; doi:10.3390/cancers15030987)
Supplement: Supplementary file 1 [file cancers-15-00987-s001.zip › cancers-2161096-supplementary.pdf]

cancers

Article

Characterization of Inorganic Scintillator Detectors for Dosimetry in Image-Guided Small Animal Radiotherapy Platforms

Ileana Silvestre Patallo [1,*], Anna Subiel [1], Rebecca Carter [2], Samuel Flynn [1,3], Giuseppe Schettino [1,4] and Andrew Nisbet [5]

Supplementary Information

Supplementary Information Figure S1

Measurements were performed in a 30 cm × 30 cm × 30 cm phantom (slabs with a total of 28 cm of Bart's WT1 solid water (Phoenix Dosimetry Ltd n.d.), with the detectors positioned at 2 cm depth. In the absence of a customized slab to hold the detector, the scintillators were sandwiched between a pair of 1 cm thick skinless water equivalent bolus material (Figure S1).

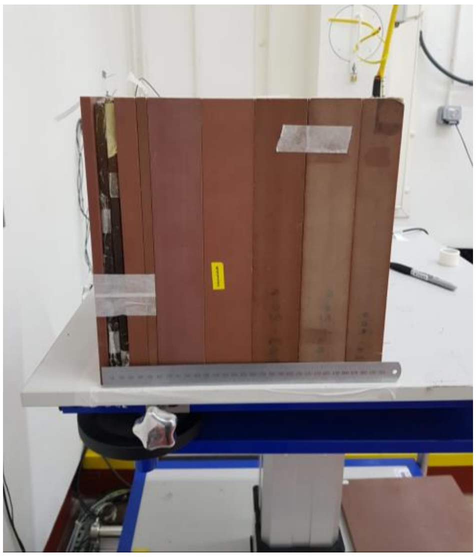

Figure S1. DoseWire cross-calibration setup: detector sandwiched between WT1 and bolus material.

Supplementary Information Figure S2

To measure variation of detector's response when its longitudinal axis was parallel to the radiation beam axis (polar angular response), a bespoke holder was used. Due to limitations with the shape and positioning of the holder in the SARRP robotic system, the gantry could only be rotated (clockwise) from -120° to 120°. Further gantry angles were not investigated as the beam would have not been able to reach the detector without interacting first with the holder.

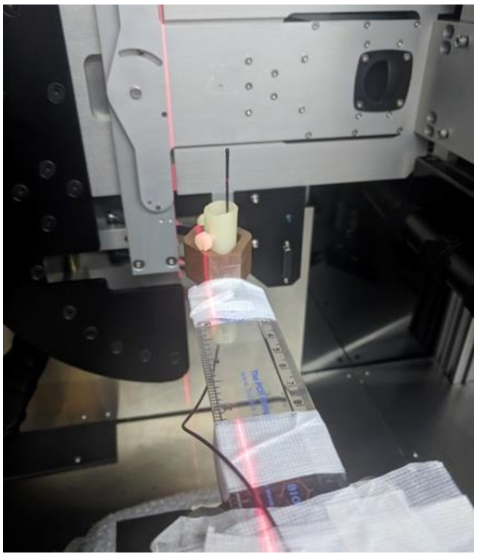

Figure S2. Setup for polar energy dependence investigation. Bespoke holder attached to the SARRP robotic positioning system.

Supplementary Information Figure S3

Graph showing linearity of the response. Each point is the average of the response of the three scintillators, normalized to the point of maximum delivered dose (correspondent to 1000 MU) and considering all reference beam qualities (HVL: 0.5, 1, 2 and 4 mm Cu). The small errors in the intercept and slope parameters of the linear fit demonstrate that linearity of the dose is independent of the beam quality.

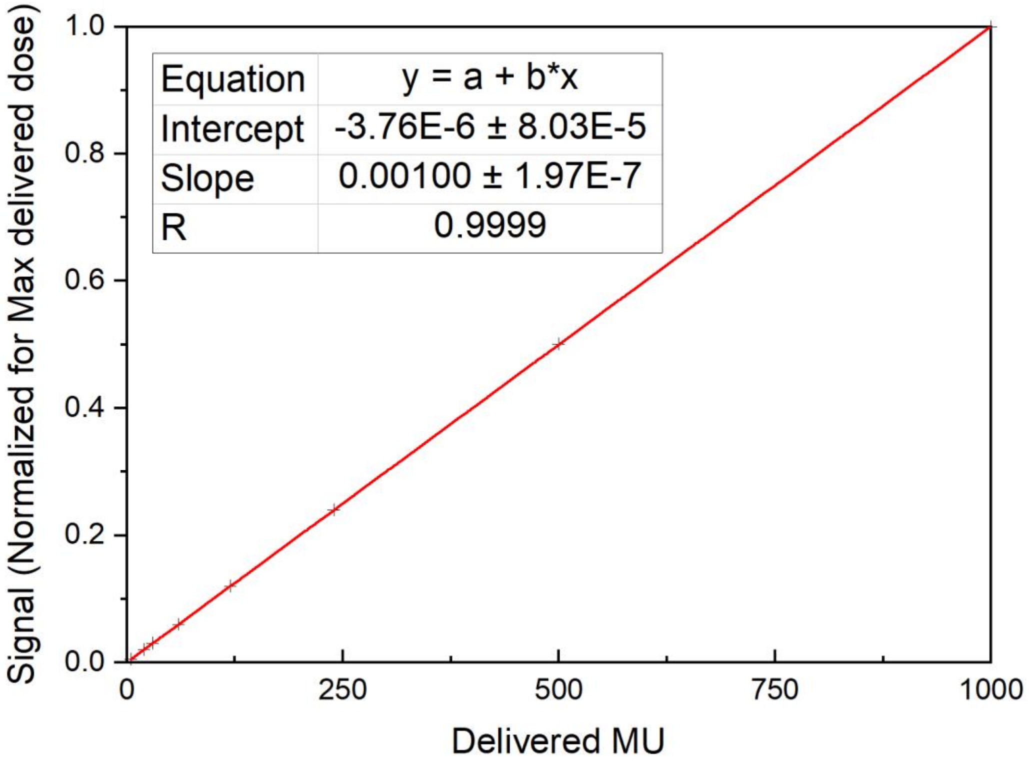

Figure S3. Linearity of response to dose for DoseWire inorganic scintillators

Supplementary Information Table S1

Tables showing data sets for calculation of the average relative output factors (ROF) for each type of detector used: DoseWire scintillator (DWS), EBT3 Gafchromic Film and metal–oxide–semiconductor detector vM1212 (Lassena CMOS).

Table S1.1 DWS ROF data sets used for the average values shown in Figure 6 of the manuscript.

		DWS					
	Eq Diam (mm)	DWS1 Set 1	DWS1 Set 2	DWS2 Set 1	DWS3 Set 1	Average	CV (%)
10mm x 10mm	11.28	1.000	1.000	1.000	1.000	1.000	0.00
5mm x 5mm	5.64	0.898	0.900	0.898	0.897	0.898	0.10
3mm x 3mm	3.39	0.855	0.858	0.856	0.850	0.855	0.33
3mm x 9mm	5.08	0.895	0.896	0.895	0.898	0.896	0.14
9mm x 3 mm	5.08	0.893	0.896	0.894	0.897	0.895	0.18
1mm Ø	1.00	0.694	0.674	0.684	0.699	0.688	1.38
0.5mm Ø	0.50	0.260	0.181	0.220	0.301	0.241	18.59

Table S1.2 EBT3 Film ROF data sets used for the average values shown in Figure 6 of the manuscript.

		EBT3 Film			
	Eq Diam (mm)	Set 1	Set 2	Average	CV (%)
10mm x 10mm	11.28	1.000	1.000	1.000	0.00
5mm x 5mm	5.64	0.940	0.925	0.932	0.84
3mm x 3mm	3.39	0.887	0.902	0.894	0.81
3mm x 9mm	5.08		0.926	0.926	0.00
9mm x 3 mm	5.08	0.918	0.917	0.918	0.07
1mm Ø	1.00	0.823	0.738	0.780	5.41
0.5mm Ø	0.50	0.532	0.490	0.511	4.07

Table S1.3 Lassena CMOS ROF data sets used for the average values shown in Figure 6 of the manuscript.

		Lassena CMOS			
	Eq Diam (mm)	Set 1	Set 2	Average	SD (%)
10mm x 10mm	11.28	1.000	1.000	1.000	0.00
5mm x 5mm	5.64	0.937	0.945	0.941	0.40
3mm x 3mm	3.39	0.902	0.890	0.896	0.65
3mm x 9mm	5.08	0.930	0.932	0.931	0.10
9mm x 3 mm	5.08	0.927	0.930	0.928	0.16
1mm Ø	1.00	0.844	0.829	0.836	0.92
0.5mm Ø	0.50	0.517	0.498	0.508	1.88

Table S1.4 Average ROF (over the three types of detectors: DSW, EBT3 Film and Lassena CMOS)

		Three Detectors	
	Eq Diam (mm)	Average	CV (%)
10mm x 10mm	11.28	1.000	0.00
5mm x 5mm	5.64	0.924	2.00
3mm x 3mm	3.39	0.882	2.15
3mm x 9mm	5.08	0.917	1.67
9mm x 3 mm	5.08	0.914	1.53
1mm Ø	1.00	0.768	7.97
0.5mm Ø	0.50	0.420	30.15
